# The phylogenetic position of dicyemid mesozoans offers insights into spiralian evolution

**DOI:** 10.1186/s40851-017-0068-5

**Published:** 2017-05-29

**Authors:** Tsai-Ming Lu, Miyuki Kanda, Noriyuki Satoh, Hidetaka Furuya

**Affiliations:** 1 Marine Genomics Unit, Okinawa Institute of Science and Technology Graduate University, Onna, Okinawa 904-0495 Japan; 2 DNA Sequencing Section, Okinawa Institute of Science and Technology Graduate University, Onna, Okinawa 904-0495 Japan; 3 Department of Biology, Graduate School of Science, Osaka University, Toyonaka, Osaka 560-0043 Japan

**Keywords:** Dicyemida, Mesozoa, Spiralia, Transcriptome, Phylogenomics, Molecular evolution

## Abstract

**Background:**

Obtaining phylogenomic data for enigmatic taxa is essential to achieve a better understanding of animal evolution. Dicyemids have long fascinated biologists because of their highly simplified body organization, but their life-cycles remain poorly known. Based on the discovery of the dicyemid *DoxC* gene, which encodes a spiralian peptide, it has been proposed that dicyemids are members of the Spiralia. Other studies have suggested that dicyemids may have closer affinities to mollusks and annelids. However, the phylogenetic position of dicyemids has remained a matter of debate, leading to an ambiguous picture of spiralian evolution.

**Results:**

In the present study, newly sequenced transcriptomic data from *Dicyema japonicum* were complemented with published transcriptomic data or predicted gene models from 29 spiralian, ecdysozoan, and deuterostome species, generating a dataset (Dataset 1) for phylogenomic analyses, which contains 348 orthologs and 58,124 amino acids. In addition to this dataset, to eliminate systematic errors, two additional sub-datasets were created by removing compositionally heterogeneous or rapidly evolving sites and orthologs from Dataset 1, which may cause compositional heterogeneity and long-branch attraction artifacts. Maximum likelihood and Bayesian inference analyses both placed *Dicyema japonicum* (Dicyemida) in a clade with *Intoshia linei* (Orthonectida) with strong statistical support. Furthermore, maximum likelihood analyses placed the Dicyemida + Orthonectida clade within the Gastrotricha, while in Bayesian inference analyses, this clade is sister group to the clade of Gastrotricha + Platyhelminthes.

**Conclusions:**

Whichever the case, in all analyses, Dicyemida, Orthonectida, Gastrotricha, and Platyhelminthes constitute a monophyletic group that is a sister group to the clade of Mollusca + Annelida. Based on present phylogenomic analyses, dicyemids display close affinity to orthonectids, and they may share a common ancestor with gastrotrichs and platyhelminths, rather than with mollusks and annelids. Regarding spiralian phylogeny, the Gnathifera forms the sister group to the Rouphozoa and Lophotrochozoa, as has been suggested by previous studies; thus our analysis supports the traditional acoeloid–planuloid hypothesis of a nearly microscopic, non-coelomate common ancestor of spiralians.

## Background

Understanding the origin and evolutionary history of metazoans has been a biological research objective for more than a century, but the phylogenetic relationships among many enigmatic taxa remain unsolved. Phylogenomic data from ambiguous taxa are essential to better comprehend animal evolution. Referring to the “new animal phylogeny,” bilaterians comprise three clades: Deuterostomia, Ecdysozoa, and Spiralia, as inferred from morphological and molecular data [1]. Earlier proposed scenarios of spiralian evolution remain contentious, possibly due to poor sampling of species belonging to certain small taxa, problematic long branches of some taxa caused by unusually fast evolution, and a paucity of morphological synapomorphies [2]. Recent phylogenomic studies have proposed that the Spiralia comprises three higher taxonomic units; i.e., that the Gnathifera (Syndermata, Gnathostomulida, and Micrognathozoa) forms a group with the clade comprising the Rouphozoa (Gastrotricha + Platyhelminthes) and the Lophotrochozoa (Mollusca, Annelida, Brachiopoda, Nemertea, etc.) [3]. However, some microscopic lineages, e.g., dicyemids, remain poorly studied, which may cause phylogenetic analyses to produce erroneous interpretations of spiralian evolution.

**Fig. 1:**
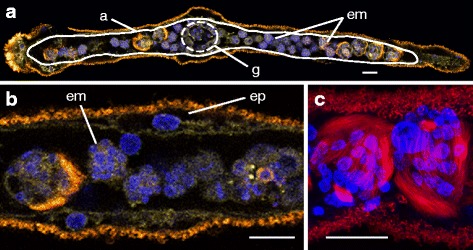
Morphology of dicyemids. **a** Dicyemid embryos (em) develop inside the central axial cell (a, *white line*), which is covered by a single layer of ciliated epidermal cells. Stained nuclei, plasma membrane, and cilia, showing the simple morphology of a sexually reproductive adult *Dicyema japonicum* with no coelom, gut, or other organs. Sperm cells and eggs produced by hermaphroditic gonads (g) fertilize, and embryos develop inside the central axial cell. Anterior is to the left. **b** The central axial cell is covered with a single layer of ciliated epidermal cells (ep). **c** Developing larvae produced sexually, possess long cilia for motility to reach a new host. Fluorescence: yellow, plasma membrane labeled with CellMask Deep Red; *blue*, nuclei labeled with DAPI; *red*, acetylated tubulin. Scale bar, 20 μm

**Fig. 2:**
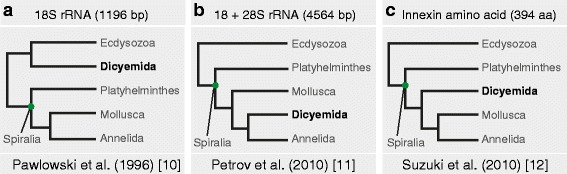
The phylogenetic position of dicyemids suggested by previous phylogenetic studies remains controversial. **a** Phylogenetic trees inferred from 18S rRNA sequences, (**b**) 18 + 28S rRNA sequences, and (**c**) innexin amino acid sequences

Dicyemids are a group of microscopic endoparasites that inhabit the renal sacs of cephalopods, mainly octopuses and cuttlefishes. They have long fascinated biologists because of their highly simplified body organization and enigmatic life-cycles [4]. Dicyemids are composed of approximately 40 cells, and they lack coeloms, circulatory systems, and other differentiated tissues (Fig. 1). In the 19th century, owing to their simple body plans, the name Mesozoa was proposed for dicyemids, as intermediates between the Protozoa (unicellular animal-like eukaryotes) and the Metazoa (multicellular animals) [5]. However, developmental studies have revealed that their embryos employ spiral cleavage, a characteristic feature of spiralians [6]. In addition, a “spiralian peptide,” which is only found in spiralian lineages, is encoded by the dicyemid *DoxC* gene; hence, thereafter, dicyemids have been regarded as degenerate triploblasts, and members of the Spiralia [7]. Moreover, a study of tool-kit genes *Pax6* and *Zic* also suggested that dicyemids are highly simplified bilaterians [8], and that their morphology might have become simplified secondarily by virtue of their parasitic lifestyles [9]. Several studies have examined the phylogenetic position of dicyemids, based on limited amounts of molecular data. Inferred from 18S rRNA sequences, dicyemids were considered related to nematodes [10] (Fig. 2a), while analyses of 18S and 28S rRNA sequences suggested a close affinity of both dicyemids and orthonectids to annelids [11] (Fig. 2b). Another study using amino acid sequences of innexin suggested that dicyemids are a sister group to the clade consisting of annelids and mollusks [12] (Fig. 2c).

Recently, large quantities of transcriptomic data from microscopic lineages have been incorporated into phylogenomic analyses, improving our understanding of spiralian evolution [2, 3]. However, dicyemids have been excluded from most recent phylogenomic studies of microscopic spiralian lineages. Here, we used genome-wide data to re-examine the phylogenetic position of dicyemids using maximum likelihood (ML) and Bayesian inference (BI) analyses. Because systematic biases sometimes occur in phylogenomic studies, thus confounding analyses due to artificial signals, we performed phylogenomic analyses using not only the complete dataset, but also sub-datasets from which rapidly evolving or compositionally heterogeneous sites had been eliminated, and from which orthologs that could have caused long-branch attraction artifacts had been excluded.

## Methods

### Sample collection, library preparation, and Illumina sequencing

A mixed life-stage sample of *Dicyema japonicum* was collected from urine in renal sacs of the host, *Octopus vulgaris*, and washed several times with artificial seawater [13]. The sample was homogenized in TRIzol Reagent (Ambion, #15596026), and RNA was extracted using the phenol-chloroform method, after which it was further purified with a QIAGEN RNeasy Micro Kit (QIAGEN, #74004). A stranded library was prepared using a NEBNext Ultra Directional RNA Library Prep Kit for Illumina (NEB, #E7420), and sequencing was performed on an Illumina HiSeq2500.

### Sequencing data sources and transcriptome assembly

Newly sequenced *D. japonicum* transcriptomic data were deposited in the DNA Data Bank of Japan (DDBJ accession number: DRA004566). Raw reads from transcriptome sequencing of *Limnognathia maerski* (SRR2131287), *Macracanthorhynchus hirudinaceus* (ERR454503), *Mesodasys laticaudatus* (SRR1797883), *Stenostomum sthenum* (SRR1801788) and *Brachionus koreanus* (SRR1658835) were downloaded from the NCBI database. Illumina raw reads were quality-trimmed with Trimmomatic (v0.33) [14]. Trimmomatic removed three bases from both ends of all reads, and deleted them once the average quality within the window fell below a threshold of 20. If reads became shorter than 36 bases, they were discarded (LEADING:3 TRAILING:3 SLIDINGWINDOW:4:20 MINLEN:36). Afterward, quality-trimmed reads were assembled *de novo* using Trinity (v2.0.6) [15] at default settings. Transcriptome assemblies of *Adineta vaga*, *Brachionus plicatilis*, *Gnathostomula paradoxa*, and *Macrodasys* sp. were adopted from the supplemental database of Struck et al. [2]. Transcriptome assemblies of *Stenostomum leucops*, *Microstomum lineare*, *Prostheceraeus vittatus*, *Geocentrophora applanata*, *Monocelis fusca*, and *Bothrioplana semperi* were adopted from Laumer et al. [3]. TransDecoder, a Trinity-plugin script, was used to extract likely coding regions within Trinity transcriptome assemblies, and transcripts were translated into amino acid sequences [16]. Protein sequences of *Tribolium castaneum*, *Drosophila melanogaster*, *Schistosoma mansoni*, and *Daphnia pulex* were downloaded from the Uniprot database. Protein sequences contributed by the *Intoshia linei* genome project [17] were downloaded from NCBI BioProject: PRJNA316116. Gene models of *Octopus bimaculatus* were downloaded from the Octopus Genome website of the Molecular Genomics Unit at OIST [18, 19]. Gene models of *Lottia gigantea* [20, 21], *Capitella teleta* [22], and *Helobdella robusta* [23] were downloaded from the JGI database.

### Compilation of datasets

Translated transcripts of all 29 taxa were assigned into ortholog groups (OGs) using a hidden Markov model-based search with HaMStR [24]. In order to minimize missing data, only OGs that contained orthologs from all 29 taxa were selected. Each OG was aligned using MAFFT (v7.220) with the Smith-Waterman algorithm and 1000 cycles of iterative refinement (--localpair --maxiterate 1000) [25]. Then alignments were trimmed using trimAl (v1.2) [26] with a gap threshold of 0.9, a similarity threshold of 0.001, and a window size of 6. Trimmed alignments shorter than 30 amino acids were discarded. Ortholog alignments were concatenated into a supermatrix using FASconCAT-G [27]. For further filtering, the gene tree of each selected ortholog was reconstructed with PhyML (v3.1) [28]. A paralog screening function of TreSpEx [29] detected possible paralogs. We use TIGER v1.2 [30] to rank sites into 20 bins based upon relative evolutionary rate of the applied dataset. Sums of branch lengths of each gene tree were determined using customized Perl scripts. An index of long-branch heterogeneity was calculated by TreSpEx (fun -e) for each gene tree. BMGE (v1.12) identified and removed the high-entropy regions and compositionally heterogeneous sites [31]. All aforementioned software was employed with default settings, unless otherwise specified.

### Contamination control

Although we washed dicyemid samples with artificial seawater several times and carefully collected individual dicyemids under a microscope, we still could not preclude the possibility of contamination with host octopus cells. In order to avoid the octopus contamination, we performed an assessment to confirm that our dicyemid transcriptome assembly was uncontaminated. We mapped 562 million Illumina raw reads from the host *Octopus vulgaris* genome back to the dicyemid transcriptome assembly using Bowtie 2 (v2.2.3) [32]. We couldn't find the octopus 18S sequence in our assembly. Only 1% of dicyemid transcripts were mapped to octopus reads, and none of the mapped transcripts were included in datasets in the present study.

### Phylogenetic reconstructions

RAxML (v8.1.20) [33] was employed to reconstruct phylogenetic trees using the maximum likelihood method with 100 bootstrap replications under the GAMMA model of rate heterogeneity. The partitioning scheme for each dataset was selected by PartitionFinderProtein v1.1.1 [34] using RAxML and relaxed clustering algorithm. For Bayesian inference analyses, a Bayesian Markov chain Monte Carlo (MCMC) sampler, PhyloBayes-MPI (v1.6j) [35] was used. For each dataset, three independent chains were calculated using a CAT + GTR mixture model. We discarded at least the first 5000 trees from each chain, and then sub-sampled every 10 trees to calculate a majority rule consensus tree of all remaining trees pooled across three chains. The PhyloBayes “bpcomp” command was used to calculate the largest discrepancy (maxdiff) observed across three independent chains. For all analyses, maxdiff values were lower than 0.15, indicating that all chains had converged. Calculated cycles and consumed time until three independent chains converged, depended on the datasets used. For instance, the analyses of Dataset 1 took seven weeks to achieve convergence using 128 cores.

### Immunostaining and imaging

Specimens for immunostaining were fixed in 4% PFA solution for 30 min, and then stored in 75% ethanol at –20°C. The immunostaining protocol was modified from a previous amphioxus study [36]. Samples were blocked for 1 hr in blocking solution (3% BSA and 0.1% Triton X-100 in PBS) and incubated in a primary antibody solution of anti-acetylated tubulin mouse monoclonal antibody (Sigma, #T6793, 1:1000 diluted in blocking solution) at 4°C overnight. Fluorescent signals were detected after incubation in a secondary antibody solution of Alexa Fluor 594-conjugated, goat anti-mouse antibody. DAPI (Invitrogen, 1 μg/mL in PBST) was used for nuclear staining, and plasma membrane were stained with CellMask (Life technology, C10046). Fluorescent images were acquired using a Zeiss 780 confocal microscope with 20× and 100× objectives.

## Results and discussion

Taking advantage of next-generation sequencing technology, phylogenomic methods utilize large molecular datasets to enable investigation of the phylogeny of animal taxa that have small body sizes, and that lack uniting synapomorphies. Newly sequenced dicyemid transcriptomic data were complemented with published transcriptomic data or predicted gene models from other spiralian, ecdysozoan, and deuterostome species. We developed a complete dataset for 29 taxa containing 348 orthologs, 58,124 amino acids, with only 6% missing data (Dataset 1). However, because large amounts of data may also amplify systematic biases, such as erroneously assigned orthologs, compositional heterogeneity, and long-branch attractions, we prepared two sub-datasets to assess the influences of potential bias sources.

Since orthologs were assigned by HaMStR [24], which sometimes groups paralogous sequences erroneously as sets of orthologous sequences, a paralog screening function of TreSpEx [29] was used to detect paralogs. After removing 17 possible paralogs from the complete dataset, two filtering criteria were used to generate Datasets 2 and 3 with potential sources of systematic bias removed. First, we obtained the sums of branch lengths of each gene tree using customized Perl scripts. If the branch length of dicyemids on a gene tree exceeded 30% of the sum of all branch lengths, this ortholog was excluded. Afterward, we ranked sites into 20 bins based upon relative evolutionary rate of the remaining alignment, and removed bin20 (the most rapidly evolving sites). That resulted in Dataset 2, containing 321 orthologs and 45,359 amino acids. For Dataset 3, an index of long-branch heterogeneity was calculated with TreSpEx for each gene tree. After excluding orthologs with index values over 100, BMGE removed the high-entropy regions and compositionally heterogeneous sites, and generated Dataset 3, with 302 orthologs and 41,202 amino acids.

**Fig. 3:**
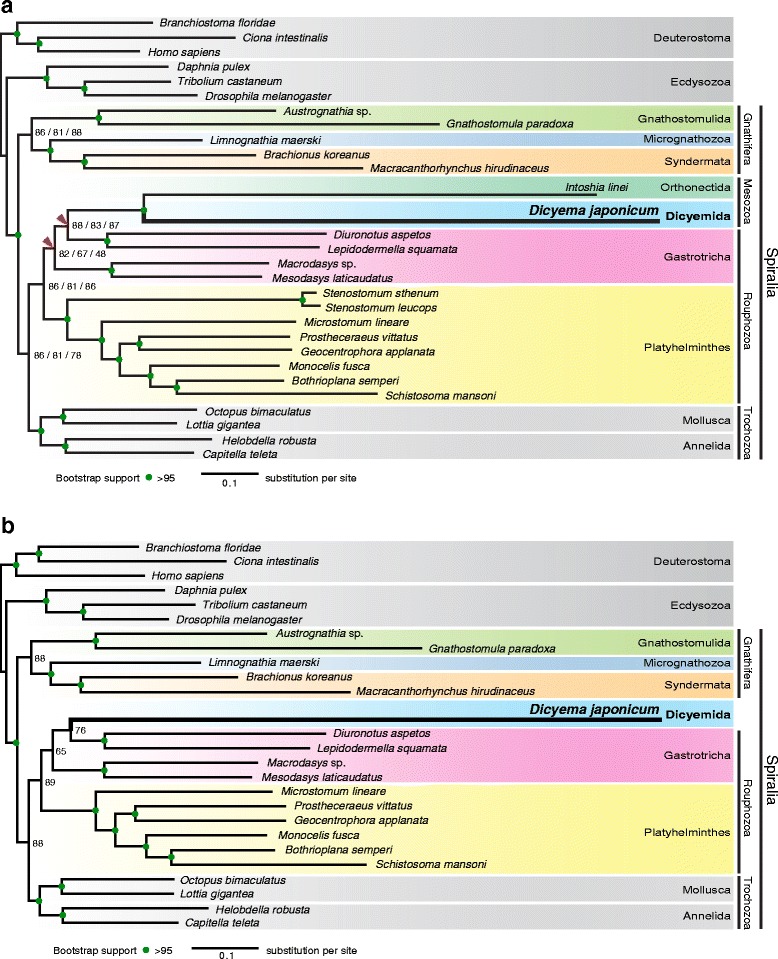
Maximum likelihood analyses suggest that the Dicyemida have a close affinity to the Orthonectida, and are nested within the Gastrotricha. **a** The maximum likelihood (ML) tree inferred from a dataset covering 29 taxa, with 348 orthologs, 58,124 amino acids, and 6% missing data. This tree topology is consistent with ML trees from analyses of two sub-datasets filtered to remove systematic biases. Analyses were executed under the GAMMA model of rate heterogeneity with 100 bootstrap replicates using RAxML. The Dicyemida displays close affinity to the Orthonectida, and both are nested within the Gastrotricha. Bootstrap values for three datasets (left to right): Datasets 1–3, respectively. Red triangles indicate different groupings from Bayesian analyses (Fig. 4). **b** ML tree, inferred from Dataset 3 covering 26 taxa for the taxon-exclusion experiment, indicates that the nesting of *D. japonicum* within the Gastrotricha probably does not reflect long-branch attraction artifacts. Filled green circles indicate >95% bootstrap support for all datasets

**Fig. 4:**
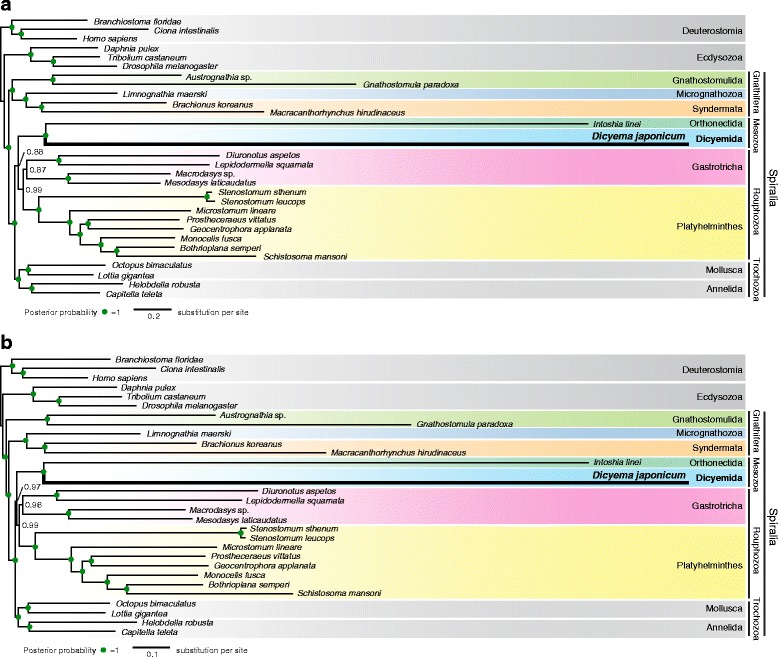
Bayesian inference analyses indicate that the Dicyemida have a closer affinity to the Orthonectida, and that the Mesozoa diverged early as a sister group to the Rouphozoa. Bayesian inference analysis was performed on three datasets, and each dataset ran three independent trains under the CAT + GTR model using PhyloBayes-MPI. Convergence of three chains occurred with a maxdiff value of <0.15. The trees inferred from Dataset 1 (**a**) and Dataset 2 (**b**) show that the Dicyemida and Orthonectida comprise a monophyletic group, sister to the Rouphozoa. Filled *green* circles indicate a posterior probability of 1

Maximum likelihood analyses were conducted using all datasets with partitioned analyses and 100 bootstrap replicates. ML trees from all three datasets showed identical topology (Fig. 3a), suggesting that our analyses were not affected by systematic bias, yet some basal splits within the Spiralia were supported with relatively mediocre bootstrap values. Bayesian inference with site-heterogeneous mixture models (CAT + GTR) is reportedly relatively resistant to long-branch attraction (LBA) artifacts [37], but it is computationally intensive. Therefore, we subjected only Datasets 1 and 2 to Bayesian analyses. BI tree topology for Dataset 1 (Fig. 4a), differed from that of Dataset 2 only in the monophyly of the Gnathifera (Fig. 4b). On BI analyses of Dataset 1, the posterior probability value was not significant for the node connecting the Mesozoa to the Rouphozoa (Fig. 4a). However, the result of Dataset 2 offered solid support for this node (Fig. 4b).

Both *D. japonicum* (Dicyemida) and *I. linei* (Orthonectida) exhibit long branches not seen in other animal taxa (Figs. 3 and 4). In all ML and BI analyses, *D. japonicum* shows a close affinity to *I. linei* with strong statistical support, although we could not categorically exclude the influence of their long-branch lengths. However, parasitic organisms often have short generation times and large population sizes, which may be associated with rapid evolution [38, 39]. The long branch length probably reflects the accelerated evolutionary rate in dicyemids [8]. It may be that dicyemids and orthonectids both evolved rapidly after diverging from a common ancestor. In addition, both dicyemids and orthonectids possess simplified morphologies without obvious synapomorphies; therefore, further comparative studies on the micro-structures or genomic features of these taxa may provide more evidence to facilitate the assessment of affinities between them.

ML analyses placed the clade Mesozoa [Dicyemida + Orthonectida] as a sister group to one gastrotrich clade [*Diuronotus aspetos* + *Lepidodermella squamata*] (Fig. 3a), and the result of a taxon-exclusion experiment based on Dataset 3 showed *D. japonicum* as nested within the Gastrotricha as well (Fig. 3b), indicating that the close relationship of *D. japonicum* with the Gastrotricha in ML analyses probably does not reflect the presence of the other long-branch taxon. Although the position of the Mesozoa does not alter across various ML analyses and is independent of potential systematic biases, the ML result for Dataset 3 (with long-branches and compositional heterogeneity removed) displayed poor bootstrap support for the root of the clade comprising dicyemids and gastrotrichs. In contrast, in BI analyses, the Dicyemida and Orthonectida formed a sister group to the Rouphozoa (Fig. 4), and BI analysis of Dataset 2 (Fig. 4b) revealed that the Mesozoa is a sister group to the Rouphozoa with significant statistical support.

A previous developmental study showed that early cleavage of dicyemids exhibits stereotypical spiral cleavage, as in the case of spiralians, such as annelids, mollusks, or platyhelminths [6]. Furthermore, studies using different molecular markers have placed mesozoans as close relatives of annelids and mollusks [10, 11]. The present phylogenomic analyses, however, offer two additional possibilities. ML analyses suggest that the Mesozoa is nested within the Gastrotricha, implying that dicyemids may be degenerate gastrotrichs. However, taking morphological traits into account, the mono-ciliated epithelial cells of gastrotrichs has been considered a diagnostic trait [40], whereas dicyemids possess multi-ciliated epithelial cells (Fig. 1c). This morphological trait barely supports the hypothesis of dicyemids nested within the Gastrotricha. Alternatively, BI trees suggest that the Mesozoa diverged early from other rouphozoans. Even so, in all analyses, the Dicyemida, Orthonectida, Gastrotricha, and Platyhelminthes constitute a monophyletic group that is a sister group to the Trochozoa (Mollusca + Annelida). This indicates that dicyemids may share characters of a common acoelomate common ancestor with gastrotrichs and platyhelminths, rather than with mollusks and annelids; nevertheless, morphological synapomorphies between dicyemids and gastrotrichs (or rouphozoans) remain to be discovered. Developing a firm grasp of spiralian evolution will still require additional developmental or genomic studies of a wide range of microscopic spiralian taxa.

Previous hypothetical scenarios of spiralian evolution remain controversial. One supports the traditional acoeloid–planuloid hypothesis of a non-coelomate common ancestor of the spiralians, whereas another suggests that the common ancestor resembled an annelid-like organism with a segmented, coelomate body plan [2]. According to the present analyses of spiralian phylogeny, the small, non-coelomate Gnathifera branched off first, and is a sister group to the Rouphozoa and Trochozoa, as reported in previous studies [2, 3]. Moreover, within the Gnathifera and Rouphozoa, most species are small, with acoelomate or pseudocoelomate body plans, whereas animals with coeloms are only found in the Lophotrochozoa (Mollusca, Annelida, etc.). The foregoing analyses and observations support the conclusion that the last common ancestor of spiralians may have been a microscopic animal lacking a coelom. It may be that microscopic lineages either maintained their ancestral morphology or that they underwent regressive evolution secondarily simplifying their morphologies, while lophotrochozoan lineages evolved more complex morphologies with a coelomic cavity and larger body size.

## Conclusions

We provide the first evidence based on a large molecular dataset that the Dicyemida has a close affinity to the Orthonectida. These taxa constitute a monophyletic group with the Gastrotricha and Platyhelminthes. Datasets with systematic biases removed (Datasets 2 and 3) show concordant results with the complete dataset (Dataset 1). The present study indicates that the mesozoan clade [Dicyemida + Orthonectida] is either a sister group to the Rouphozoa or nested within the Gastrotricha in BI and ML analyses, respectively, but further genomic and developmental studies will be necessary to examine these competing hypotheses. Nonetheless, the present results agree with earlier findings that the Gnathifera branched off first, and comprise a sister group to the Rouphozoa and Lophotrochozoa, supporting the previously proposed acoeloid-planuloid hypothesis of a nearly microscopic non-coelomate common ancestor of the spiralians.
